# Notes and News

**Published:** 1895-11-23

**Authors:** 


### NOTES AND NEWS.

(Collected and Communicated.)

The Prince of Wales has consented to preside at a festival dinner in aid of the funds of Guy's Hospital in May next.

Mr. W. G. Farrance Bosworth, the secretary's clerk of St. Mary's Hospital, has been appointed secretary of the North London Hospital for Consumption, Hampstead, in succession to Mr. Lionel F. Hill, M.A., who is about to retire. This is the second secretarial appointment secured during the past six months by members of the staff of St. Mary's.

The Chairman of the Middlesex Hospital is issuing an appeal for more annual subscribers for that institution. The expenditure amounts to £30,000, whilst annual subscriptions only amount to £3,000 per annum. At the present time funds are especially needed, as the hospital is building a most useful addition in the shape of a new cancer wing, and establishing a convalescent home.

English people are apt to look with a certain amount of complacency on the fact that all the world is occupied in feeding them. It will not do, however, to be blind to the evils which accompany this importation of food, and especially of live creatures destined for food, from the far corners of the earth. The revelations recently made regarding the horrors connected with the traffic in live cattle from Australia ought to be sufficient to show that such a trade, likely as it is to entail additional difficulties on our agricultural population, ought to be conducted under the strictest supervision in regard to the treatment of the animals, even although thereby the expense of transit may be increased. Not only is it immoral that we should sanction in others a course of cruelty and ill-treatment of cattle which we would not for a moment permit among the drovers in our own country, bad as the condition of our markets may sometimes be; but it becomes ridiculous when it is seen that we are thereby searing our consciences for the benefit of a foreign trade by which our own farmers run a considerable chance of being ruined.

A Convalescent Home for female patients has been opened at Murthly, in Scotland, in connection with the asylum there.

Dr. C. S. Sherrington has resigned the post of Demonstrator of Physiology at St. Thomas's Hospital, and Dr. J. G. Brodie has been appointed in his place. Dr. Brodie previously held the same appointment at the London Hospital.

The inhabitants of Guildford and adjoining district are bringing an action against the Guildford, Godalming, and Woking Joint Hospital Board to prevent them using a site they have acquired for the erection of a small-pox hospital, which, it is contended, would be situated too near dwelling houses.

The Hospital Saturday Fund, which has received £16,000 in contributions in 1895, will remain open until the end of the year. A special appeal has been made to the various workshops and business houses of the metropolis for increased support, and it is hoped that a £20,000 collection will be reached before the list closes.

At a cost of £11,000, an infectious hospital for the borough of Burton has been erected. It consists of an administrative block, an isolation block, a scarlet fever pavilion, laundry block, mortuary, and discharging rooms, bath-room for discharged patients, and entrance lodge and waiting-room. The buildings are of brick, with pressed brick facings, and stone sills and dressings. The roofing is of green Westmoreland slate. Great care has been bestowed in construction and fitting.

A cottage hospital has been opened at Porth, in Wales. Colonel Picton Turberville gave the site, and the institution, which cost £3,000, is opened free of debt. The building is of local stone, with red brick dressings. It will accommodate eight patients on the ground floor, and two wards on the first floor are reserved for private patients or cases requiring isolation, we are informed, though we trust a more definite rule will be observed in practice as to the use to which they are to be put.

The curative treatment of habitual drunkards is likely to be taken up by the Government in Austria. It is proposed to establish retreats, where inmates shall be received for two years. They may enter of their own accord or be placed there under compulsion. If under compulsion a legal trial must be previously held. The period of retention may be curtailed to one year, the patient being released on a system resembling that of ticket of leave. If a patient is shown to be incurable he may be retained for life.

The Metropolitan Public Gardens Association, under the presidency of the Earl of Meath, who takes the keenest personal interest in its labours, is carrying out a work of national importance in preserving open spaces for the benefit of Londoners. Bartholomew-square, Old-street, St. Luke's, was opened publicly by Lord Meath a few weeks since, and a lease of this ground has been granted by the governors of St. Bartholomew's Hospital, at a nominal rental, for a term of eighty years. More such open spaces are sadly needed in the crowded district of St. Luke's, the acquisition of which will no doubt be brought about in time through the persevering efforts of this association.

